# Functions and regulation of the MRX complex at DNA double-strand breaks

**DOI:** 10.15698/mic2016.08.517

**Published:** 2016-07-27

**Authors:** Elisa Gobbini, Corinne Cassani, Matteo Villa, Diego Bonetti, Maria P. Longhese

**Affiliations:** 1 Dipartimento di Biotecnologie e Bioscienze, Università di Milano-Bicocca, Piazza della Scienza 2, 20126 Milan, Italy.; 2 Institute of Molecular Biology gGmbH (IMB), 55128 Mainz, Germany.

**Keywords:** double-strand break, resection, MRX, nucleases, Tel1, Rif2, Sae2

## Abstract

DNA double-strand breaks (DSBs) pose a serious threat to genome stability and cell survival. Cells possess mechanisms that recognize DSBs and promote their repair through either homologous recombination (HR) or non-homologous end joining (NHEJ). The evolutionarily conserved Mre11-Rad50-Xrs2 (MRX) complex plays a central role in the cellular response to DSBs, as it is implicated in controlling end resection and in maintaining the DSB ends tethered to each other. Furthermore, it is responsible for DSB signaling by activating the checkpoint kinase Tel1 that, in turn, supports MRX function in a positive feedback loop. The present review focuses mainly on recent works in the budding yeast Saccharomyces cerevisiae to highlight structure and regulation of MRX as well as its interplays with Tel1.

## INTRODUCTION

DNA double-strand breaks (DSBs) are cytotoxic lesions that threaten genomic integrity. Misrepair of DSBs often leads to chromosome rearrangements and loss of genetic information that can result in cell death or oncogenic transformation. DSBs can arise spontaneously in eukaryotic cells either during DNA replication or as intermediates in programmed recombination events, such as meiosis and immune system development. They can also be induced by exposure to DNA-damaging agents used in cancer therapies, including ionizing radiation and topoisomerase poisons.

Cells have evolved two main mechanisms to repair DSBs: non-homologous end joining (NHEJ) and homologous recombination (HR). NHEJ directly religates the two broken DNA ends with no or minimal base pairing at the junction 1. HR uses intact homologous duplex DNA sequences (sister chromatids or homologous chromosomes) as templates for repairing DSBs in an error-free manner 2,3. In order to repair DSBs by HR, the 5’ strands of each DSB end has to be nucleolytically degraded, in a process referred to as resection 4,5. The resulting 3’-ended single-stranded DNA (ssDNA) tails are first coated by the ssDNA binding complex Replication Protein A (RPA), which is then replaced by the recombinase Rad51 to form a right-handed helical filament that searches for DNA homologous sequences and catalyzes invasion of the duplex DNA molecules 2,3. Initiation of resection not only channels DSB repair to HR but irreversibly inhibits NHEJ, indicating that this process is critical for discriminating between homology-dependent and end-joining repair of DSBs.

The highly conserved MRX/MRN complex (Mre11-Rad50-Xrs2 in yeast; Mre11-Rad50-Nbs1 in mammals) is among the first protein complexes that are recruited at DSBs 6. MRX plays an important role in controlling end resection and in maintaining the DSB ends tethered to each other for their repair by NHEJ or HR 4,5,7. Furthermore, it is implicated in the recruitment and activation of the protein kinase Tel1 (ATM in mammals), which plays an important role in DSB signaling 8. Finally, MRX is essential to generate and resect meiotic DSBs, which are created by the Spo11 transesterase that forms a covalent linkage between a conserved tyrosine residue and the 5’ end of the cleaved strand 9. Here, we review structure and regulation of the MRX complex, as well as its crosstalk with Tel1, focusing mainly on budding yeast *S. cerevisiae*, where the most mechanistic information is available.

## ARCHITECTURE OF THE MRX COMPLEX

**Figure 1:**
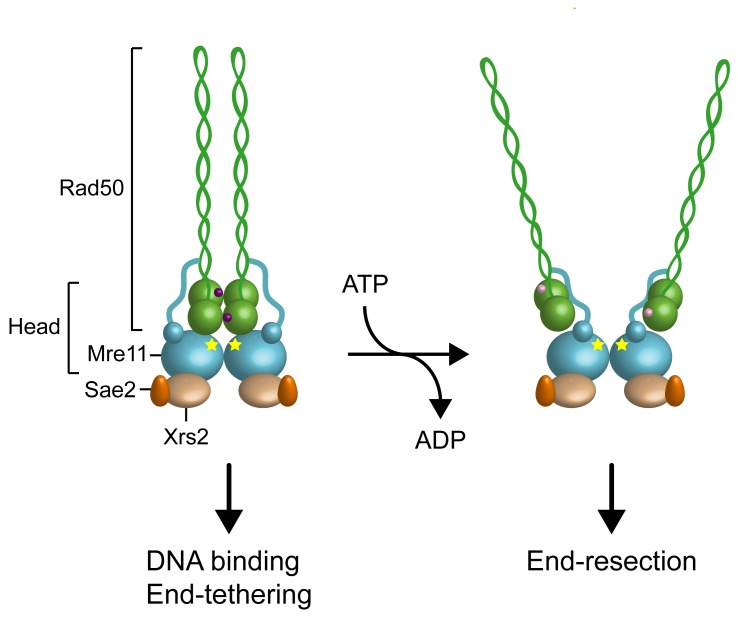
FIGURE 1: Structural organization of the MRX complex. The ATP-bound state of Rad50 negatively regulates MRX nuclease activity by masking the Mre11 nuclease sites. ATP hydrolysis by Rad50 causes conformational changes of both Rad50 and Mre11, resulting in disengagement of Rad50 dimer and exposure of the Mre11 active sites that can access DNA to initiate DSB resection. The Mre11 nuclease sites are indicated by yellow stars. ATP and ADP are indicated by purple and pink dots, respectively.

The Mre11 subunit of MRX contains five conserved phosphoesterase motifs that are essential for both 3’-5’ double-stranded DNA (dsDNA) exonuclease and ssDNA endonuclease activities 10,11,12,13,14,15,16. Mre11 interacts with Rad50, whose domain organization is similar to that of the structural maintenance of chromosomes (SMC) family of proteins 17. Rad50 is an ATPase belonging to the ABC ATPase superfamily that is characterized by the ATP-binding motives Walker A and B located at the amino- and carboxy-terminal regions of the protein. These motives associate together, with the intervening sequence forming a long antiparallel coiled-coil. Two Rad50 ATPase domains interact with two Mre11 nuclease proteins to form a “head” domain, which is the DNA binding and processing core of MRX 18,19,20,21,22 (Fig. 1). The Rad50 coiled-coil region protrudes from the head domain and its base interacts with two α helices of Mre11 located carboxy-terminal to the nuclease core domain 23,24,25,26. At the apex of the coiled-coil, where the N-terminal and C-terminal regions fold back on themselves, a CXXC motif creates a zinc-mediated hook that allows dimerization between Rad50 molecules within a dimeric assembly (intra-complex) or between Rad50 molecules in separate dimeric assemblies (inter-complex). The ability of Rad50 to dimerize with Rad50 molecules that are bound to different DNA ends by Zn-dependent dimerization of the hook domains can account for the end-tethering activity of the MRX complex 27,28,29,30,31,32.

While Mre11 and Rad50 are conserved in bacteria and archaea, only eukaryotes possess Xrs2 (Nbs1 in mammals), which is responsible for nuclear localization of Mre11 33. The addition of a nuclear localization signal to Mre11 can partially suppress the hypersensitivity to DNA damaging agents of Xrs2-deficient cells 33, suggesting that localization of Mre11 into the nucleus is one of the main functions of Xrs2. Xrs2/Nbs1 contains a variety of protein-protein interaction modules, among which there is a conserved region within the C-terminus that is responsible for the interaction with Tel1/ATM 34,35.

## FUNCTIONAL DYNAMICS OF THE MRX COMPLEX 

The MRX complex plays a central role in signaling, processing and repairing of DSBs. Structural studies have shown that these diverse functions are regulated by the ATP binding and hydrolysis activity of Rad50 that induce conformational changes of both Rad50 and Mre11. In the presence of ATP, Mre11 and Rad50 adopt a “closed” conformation, in which Rad50 head domains dimerize and occlude the nuclease active site of Mre11 (Fig. 1) 24,26. ATP hydrolysis drives the rotation of the two nucleotide binding domains of Rad50, leading to disengagement of the Rad50 dimer and DNA melting, so that the Mre11 active sites can access DNA to initiate DSB resection 23,36 (Fig. 1). Point mutations that stabilize the ATP-bound conformation of Rad50 result in both reduced Mre11 nuclease activity and increased DNA binding and end-tethering 37. By contrast, mutations that increase ATP hydrolysis enhance both Mre11 nuclease and end-resection activities 37. These data suggest that the ATP-bound state is required for DNA binding and tethering, whereas release from the ATP-bound state by ATP hydrolysis is necessary to allow access to DNA of the Mre11 nuclease active site and subsequent DSB resection (Fig. 1).

Rad50 has a slow ATP hydrolysis rate 38, suggesting that either MRX exists mostly in the ATP-bound state or other proteins can promote ATP hydrolysis within the cell. In *S. cerevisiae*, MRX is known to interact with Rif2, which is recruited to telomeric DNA ends by Rap1 and negatively regulates telomerase action 39,40,41,42. Rif2 was recently found to be recruited also to intrachromosomal DSBs in a manner partially dependent on MRX 43. Interestingly, Rif2 enhances the ATP hydrolysis activity of the MRX complex 43. Furthermore, *rif2*∆ cells show an increased efficiency of both end-tethering and NHEJ compared to wild type cells, indicating that Rif2 counteracts end-tethering and DSB repair by NHEJ 43. These observations suggest that Rif2 can modulate the choice between HR and NHEJ by promoting the transition of the MRX complex from a closed state, required for tethering, to an open state that is competent for DSB resection. Interestingly, Rif2 is known to counteract NHEJ at telomeres 44. Whether this Rif2 function depends on a Rif2-mediated regulation of MRX conformational changes is an interesting question that remains to be addressed.

## MRX IN END-RESECTION

The first step in HR is the degradation of the 5’ DNA strands on either side of the DSB through a process termed resection 4,5. Several lines of evidence indicate that the MRX complex functions together with the Sae2 protein (CtIP in mammals) in the processing of the DSB ends. In fact, *S. cerevisiae* mutants lacking Sae2 or any component of the MRX/MRN complex delay resection of an endonuclease-induced DSB by acting in the same epistasis group 45,46,47,48. Furthermore, both Sae2 and MRX play a unique role in the processing of hairpin-containing DNA structures 49,50,51. Finally, *sae2*∆, *mre11* nuclease defective mutants and a class of separation-of-function *rad50* and *mre11* alleles, named *rad50s *and* mre11s*, allow formation of meiotic DSBs but prevent their resection 11,52,53,54,55,56.

**Figure 2:**
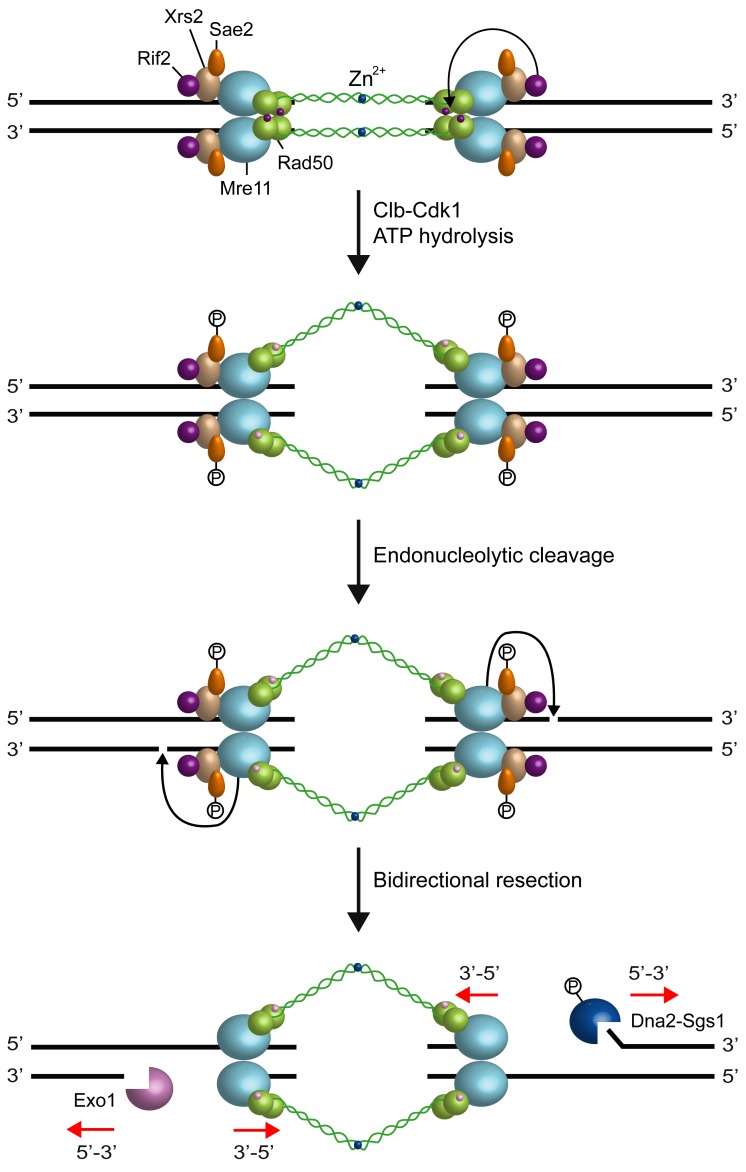
FIGURE 2: **Model for DSB resection.** The MRX complex and Sae2 are recruited to DNA ends. In the ATP-bound state, Rad50 blocks the Mre11 nuclease and MRX promotes DNA tethering. After ATP hydrolysis by Rad50, the Mre11 nuclease sites are exposed and can catalyze an endonucleolytic cleavage of the 5’ strand. Rif2 can promote the ATP hydrolysis activity of Rad50. MRX-mediated incision requires Sae2 phosphorylation by Cdk1-Clb and allows bidirectional processing by Exo1 and Sgs1-Dna2 in the 5’-3’ direction from the nick and by MRX in the 3’ to 5’ direction toward the DSB ends. ATP and ADP are indicated by purple and pink dots, respectively.

However, Mre11 has a 3’-5’ dsDNA exonuclease activity, whose polarity is opposite to that required to generate the 3’-ended ssDNA at the DSB ends 12,13. Mre11 possesses also a weak endonuclease activity on 5’-terminated dsDNA strands 16,57,58, which is strongly stimulated by Sae2 and by a protein block at the DNA ends 59. Furthermore, *sae2∆* and *mre11* nuclease mutants are defective in the removal of covalent adducts, such as Spo11 (the transesterase that generates meiotic DSBs and remains covalently bound to the 5’ strands of the ensuing breaks) 60,61,62,63 or hairpin-containing DNA structures, from DNA ends 49,50,51. The same mutants are also hypersensitive to both camptothecin, which extends the half-life of DNA-topoisomerase cleavage complexes, and ionizing radiations, which can generate chemically complex DSBs 64,65,66. Altogether, these observations suggest that the MRX complex initiates DNA resection by creating a nick that provides an internal entry site for nucleases capable of degrading DNA in a 5’-3’ direction (Fig. 2).

These nucleases comprise the 5’-3’ exonucleases Exo1 and the endonuclease Dna2, which control two partially overlapping pathways 67,68. While Exo1 is able to release mononucleotide products from a dsDNA end 69,70,71, Dna2-mediated resection needs the RecQ helicase Sgs1 (BLM in humans) that unwinds double-stranded DNA in a 3’-5’ polarity 72,73,74. Noteworthy, the MRX complex not only provides an entry site for Dna2 and Exo1, but it has also a structural role in allowing their recruitment to the DSB 75, thus explaining why *mre11*∆ cells show a resection defect more severe than *sae2*∆ or *mre11 *nuclease defective mutants. In any case, *mre11*∆ cells are severely defective in DSB resection when the break is present in the G2 phase of the cell cycle, whereas they slow down resection only of two fold when the break occurs when they are exponentially growing 45,46,47,48. This observation, together with the finding that a DSB is processed more efficiently during S phase than in G2 76, suggests that ongoing DNA replication can partially bypass MRX requirement in DSB resection. 

Both Sae2 and Dna2 have been shown to be targets of cyclin-dependent kinase (Cdk1 in yeast)-Clb complexes 77,78, which allow DSB resection to take place only during the S and G2 phases of the cell cycle when sister chromatids or homologous chromosomes are present as repair templates 79,80. Substitution of Sae2 S267 with a nonphosphorylatable residue impairs DSB processing, whereas the same process takes place when Sae2 S267 is replaced by a residue mimicking constitutive phosphorylation 77. Similarly, substitution of three Cdk1 consensus sites of Dna2 with alanines reduces extensive resection 78. Notably, the lack of any subunit of the Ku complex allows DSB resection in G1-arrested cells when Cdk1-Clb activity is low 76,81, suggesting that Cdk1-Clb activity can relieve the inhibitory effect exerted by Ku on Sae2. The finding that this resection in G1-arrested Ku-deficient cells is limited to the break-proximal sequence 76,81 indicates that Cdk1 is still required to activate proteins involved in extensive DSB resection such as Dna2. 

Altogether, these data support a model, where MRX is recruited to the DSB ends in the ATP-bound state. In this configuration, MRX maintains the DSB ends tethered to each other to allow DSB repair by NHEJ (Fig. 2). Upon ATP hydrolysis by Rad50 and Cdk1-mediated phosphorylation events, MRX and Sae2 provide an initial incision of the 5’ strand at a certain distance from the DSB end. As first proposed by Garcia *et al.* 82, this initial cleavage is followed by bidirectional resection using the Mre11 3’-5’ exonuclease and the 5’-3’ nuclease activities of Exo1 or Dna2-Sgs1 (Fig. 2).

## ACTIVATION OF Tel1 BY MRX

In addition to promoting end-resection and to maintaining the DSB ends tethered to each other, MRX/MRN is required to activate Tel1, which is a member of the evolutionary conserved phosphoinositide 3-kinase-related protein kinase (PIKK) family and whose mammalian ortholog is called ATM (Ataxia Telangiectasia Mutated) 8. The PIKK enzymes are large serine/threonine protein kinases characterized by N-terminal HEAT repeat domains and by C-terminal kinase domains 83. The C-terminal kinase domain of ATM is flanked by two regions called FAT (FRAP, ATM, TRRAP) and FATC (FAT C-terminus), which both participate in the regulation of the kinase activity 84. 

Tel1/ATM is a master regulator of the DNA damage response in both yeast and mammals, where it coordinates checkpoint activation and DNA repair in response to DNA DSBs and oxidative stress 85,86. Biallelic mutations in ATM result in ataxia telangiectasia (AT), an autosomal recessive inherited disease characterized by cerebellar degeneration, strong predisposition to malignancy, growth retardation, radiosensitivity, immune deficiencies, and premature aging 87,88,89. The functional interaction between MRX/MRN and Tel1/ATM is supported by the finding that biallelic mutation in the *MRE11* gene causes a genetic syndrome, called ataxia-telangiectasia-like disease (ATLD), whose clinical phenotypes are nearly indistinguishable from AT 90,91. ATLD cells exhibit reduced activation of ATM by DSBs, suggesting that MRN is required for optimal ATM activation following DSB induction, thus explaining the AT-like phenotype of ATLD patients. 

**Figure 3:**
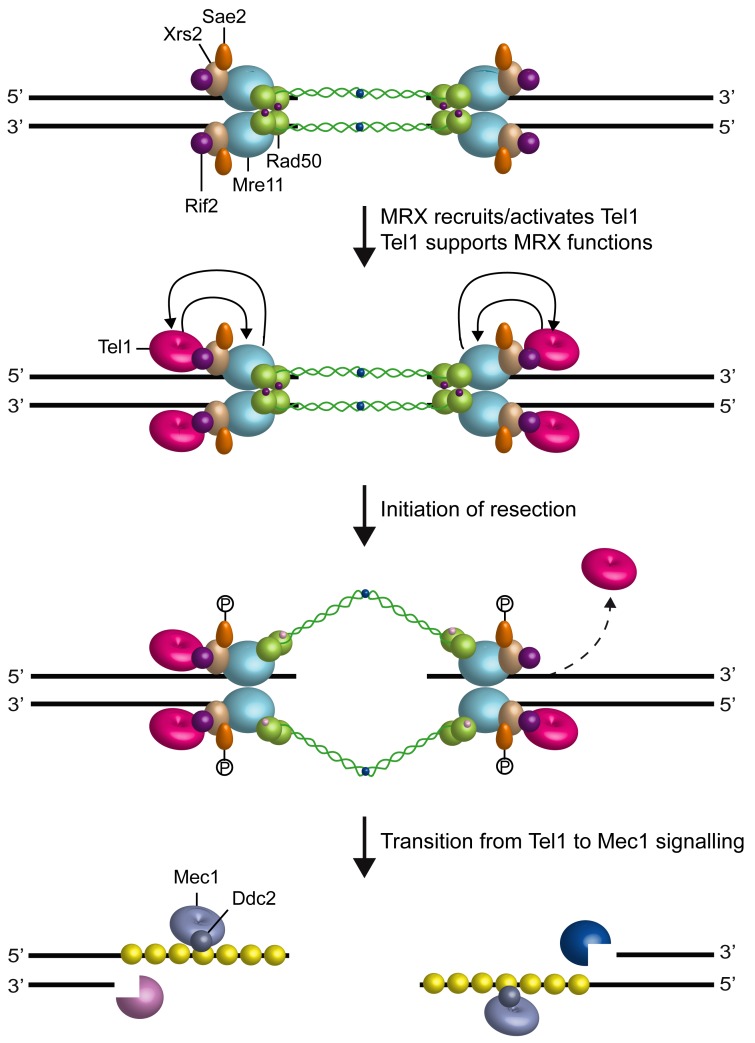
FIGURE 3: Crosstalk between MRX and Tel1. The MRX complex is required to recruit and activate Tel1, which initiates DSB signaling. Tel1, once loaded to the DSB ends by MRX, supports MRX function by promoting its association to the DSBs ends. Rif2 counteracts Tel1 recruitment to DSBs by competing with Tel1 for binding to MRX and stimulates Rad50 ATPase activity. Initiation of DSB resection by MRX-Sae2, Exo1 and Sgs1-Dna2 generate 3’-ended ssDNA tails that promotes a switch from a dsDNA-Tel1 to a ssDNA-Mec1 signaling activity.

Subsequent studies have revealed that MRX/MRN drives the localization of Tel1/ATM to the site of damage through direct interaction between Tel1/ATM with Xrs2/Nbs1 35,92,93,94 (Fig. 3). The Tel1 kinase activity is stimulated by MRX binding to DNA-protein complexes at DSBs 95 and the purified MRX/MRN complex increases the catalytic activity of Tel1/ATM in the presence of DNA fragments 96,97,98, suggesting that MRX/MRN also controls Tel1/ATM catalytic activity through an unknown mechanism.

Tel1 was originally identified in *S. cerevisiae* by screen for genes involved in telomere length maintenance 99,100,101. In addition to its role in DSB repair, Tel1 is required to maintain telomere length by promoting telomerase recruitment through phosphorylation events 102. Deletion of any subunit of the MRX complex causes telomere shortening similar to that caused by the lack of Tel1 or both Tel1 and Rad50, indicating that Tel1 acts in the same pathway of MRX in telomere length maintenance 103. As it is observed at DSBs, Tel1 binding to telomeres is dependent on an interaction between Tel1 and the carboxyl terminus of the Xrs2 subunit of the MRX complex 104,105. Tel1 association to telomeres is counteracted by Rif2, which is known to inhibit telomerase-dependent telomere elongation 39,40. Co-immunoprecipitation experiments show that the C terminus of Xrs2 interacts with Rif2 41. As Tel1 also binds this Xrs2 region 41, Rif2 may reduce Tel1 association to telomeres by interfering with MRX-Tel1 interaction. Further support for a Tel1-Rif2 competition comes from the recent finding that a hypermorphic allele of *TEL1* (*TEL1-hy909*) is capable to overcome the inhibitory activity of Rif2 on MRX 42. The Rif2 function in modulating MRX-Tel1 interaction is not restricted to telomeres. In fact, the lack of Rif2 increases the association of MRX also to intrachromosomal DNA ends in a Tel1-dependent manner 43 (Fig. 3). Consistent with a direct role of this protein at DSBs, Rif2 can bind DNA ends both *in vitro* and *in vivo* 43, although the amount of Rif2 bound at a DSB flanked by telomeric repeats is higher than that found at a DSB that does not contain telomeric sequences 41,42,43. 

In *S. cerevisiae*, Tel1 signaling activity is disrupted when the DSB ends are subjected to 5’-3’ exonucleolytic degradation 106 (Fig. 3). Similarly, ATM activation in mammals is triggered by blunt ends or short overhangs and is inhibited by long overhangs of 3’ or 5’ ssDNA 107. Interestingly, the lack of either Sae2 or Mre11 nuclease activity enhances Tel1/ATM activation at DSBs by increasing MRX persistence at DSBs 47,108, suggesting that Sae2 and Mre11 nuclease can inhibit Tel1/ATM signaling activity. As the mammalian counterpart of MRX has been shown to bind ssDNA-dsDNA junctions 109, the slowing down of DSB resection might lead to MRX persistence at DSBs by increasing the stability of these DNA structures. Alternatively, ssDNA near the dsDNA junction may break to form a second DSB that activates MRX-Tel1, as suggested in 110. 

Attenuation of Tel1/ATM signaling by nuclease-mediated DSB resection is accompanied by activation of the checkpoint kinase Mec1 (ATR in mammals), which is another member of the phosphoinositide 3-kinase-related protein kinase family 8 (Fig. 3). In both yeast and mammals, all Mec1/ATR functions at DSBs depend on the interaction with Ddc2 (ATRIP in mammals), which mediates Mec1/ATR recruitment on RPA-coated ssDNA 3’ overhangs 111,112,113,114. 

## REGULATION OF MRX BY Tel1

Recent data have shown that Tel1, once recruited to DSBs by MRX, supports MRX function in a positive feedback loop (Fig. 3). In fact, a screen for *S. cerevisiae* mutants that require Tel1 to survive to genotoxic treatments recently showed that a mutation in Rad50 (*rad50-V1269M*) makes *tel1*∆ cells hypersensitive to DNA damaging agents 43. The *rad50-V1269M* mutation impairs MRX association at DSBs that is further reduced by the lack of Tel1, indicating that Tel1 promotes/stabilizes MRX association to the DSB. Interestingly, Tel1 exerts this function independently of its kinase activity 43, suggesting that it plays a structural role in promoting/stabilizing MRX retention to DSBs. Similarly, the lack of Tel1, but not of its kinase activity, was shown to impair MRX association also at DNA ends flanked by telomeric DNA repeats 43.

Although the *rad50-V1269M* mutation resides in the globular domain of Rad50, *rad50-V1269M* *tel1*∆ cells are severely defective in the maintenance of the DSB ends tethered to each other 43, suggesting that the Rad50 hook and globular domains function interdependently. Consistent with this hypothesis, it has been shown that proper association to DNA of the globular domain can induce parallel orientation of the Rad50 coiled-coil domains that favours intercomplex association needed for DNA tethering 22. Furthermore, mutations in the hook domain that reduce its dimerization state affect the MRX functions specified by the globular domain, including Tel1/ATM activation, Rad50-Mre11 interaction, NHEJ and DSB resection 115. Altogether these results support a model wherein Tel1, once loaded at DSBs by MRX, exerts a positive feedback by promoting an end-specific association of MRX with DNA (Fig. 3). This Tel1-mediated regulation of DNA-MRX retention is important to allow the establishment of a productive MRX intercomplex association that is needed to maintain DNA ends in close proximity.

Defects in maintaining the DSB ends tethered to each other in *tel1*∆ *rad50-V1269M* cells affect DSB repair not only by NHEJ, but also by HR 43. During HR, the 5’ ends at the DSB are degraded to yield 3’ ssDNA tails that invade an intact homologous duplex DNA to prime DNA synthesis. After ligation of the newly synthesized DNA to the resected 5’ strands, a double Holliday junction intermediate is generated and can be resolved by endonucleolytic cleavage to produce non-crossover (NCO) or crossover (CO) products. However, in mitotic cells, the invading strand is often displaced after limited synthesis and anneals to the 3’ ssDNA tail at the other end of the DSB. After fill-in synthesis and ligation, this mechanism, called synthesis-dependent strand annealing (SDSA), generates exclusively NCO products and explain the lower incidence of associated COs during mitotic DSB repair 116,117,118. Interestingly, *tel1*∆ *rad50-V1269M* cells are specifically impaired in SDSA 43, suggesting that the tethering activity exerted by MRX can be particularly important to promote the annealing of the displaced strand to the 3’ ssDNA tail at the other end of the DSB. By contrast, this function can be escaped when the second DSB end is already captured by the D-loop and the DNA intermediate is stabilized by the formation of a double Holliday junction.

## CONCLUSION

Overall, the MRX complex emerges as a multifunctional enzyme involved in a number of activities that include sensing and processing of the DSB ends. Furthermore, its association with Tel1 links DSB sensing with signaling by the checkpoint machinery to coordinate DSB repair with cell cycle progression. These functions are regulated by ATP binding and hydrolysis activities of Rad50 that provide intrinsic dynamics and flexibility properties. Furthermore, the Tel1 protein itself, once recruited at DNA ends by MRX, supports the end-tethering activity of the MRX complex by facilitating its proper association to DNA ends. These observations reveal the complex architecture that characterizes the activity of MRX and Tel1 in DNA-damage response, maintenance of genetic stability and cell cycle regulation.

## References

[1] Chiruvella KK, Liang Z, Wilson TE (2013). Repair of double-strand breaks by end joining.. Cold Spring Harb Perspect Biol.

[2] San Filippo J, Sung P, Klein H (2008). Mechanism of eukaryotic homologous recombination.. Annu Rev Biochem.

[3] Mehta A, Haber JE (2014). Sources of DNA double-strand breaks and models of recombinational
DNA repair.. Cold Spring Harb Perspect Biol.

[4] Longhese MP, Bonetti D, Manfrini N, Clerici M (2010). Mechanisms and regulation of DNA end resection.. EMBO J.

[5] Symington LS (2014). End resection at double-strand breaks: mechanism and
regulation.. Cold Spring Harb Perspect Biol.

[6] Lisby M, Barlow JH, Burgess RC, Rothstein R (2004). Choreography of the DNA damage response: spatiotemporal
relationships among checkpoint and repair proteins.. Cell.

[7] Stracker TH, Petrini JH (2011). The MRE11 complex: starting from the ends.. Nat Rev Mol Cell Biol.

[8] Gobbini E, Cesena D, Galbiati A, Lockhart A, Longhese MP (2013). Interplays between ATM/Tel1 and ATR/Mec1 in sensing and signaling
DNA double-strand breaks.. DNA Repair.

[9] Lam I, Keeney S (2014). Mechanism and regulation of meiotic recombination
initiation.. Cold Spring Harb Perspect Biol.

[10] Bressan DA, Olivares HA, Nelms BE, Petrini JH (1998). Alteration of N-terminal phosphoesterase signature motifs
inactivates Saccharomyces cerevisiae Mre11.. Genetics.

[11] Furuse M, Nagase Y, Tsubouchi H, Murakami-Murofushi K, Shibata T, Ohta K (1998). Distinct roles of two separable in vitro activities of yeast
Mre11 in mitotic and meiotic recombination.. EMBO J.

[12] Paull TT, Gellert M (1998). The 3' to 5' exonuclease activity of Mre11 facilitates repair of
DNA double-strand breaks.. Mol Cell.

[13] Trujillo KM, Yuan S-SF, Lee EY-HP, Sung P (1998). Nuclease activities in a complex of human recombination and DNA
repair factors Rad50, Mre11, and p95.. J Biol Chem.

[14] Usui T, Ohta T, Oshiumi H, Tomizawa J, Ogawa H, Ogawa T (1998). Complex formation and functional versatility of Mre11 of budding
yeast in recombination.. Cell.

[15] Moreau S, Ferguson JR, Symington LS (1999). The nuclease activity of Mre11 is required for meiosis but not
for mating type switching, end joining, or telomere
maintenance.. Mol Cell Biol.

[16] Trujillo KM, Sung P (2001). DNA structure-specific nuclease activities in the Saccharomyces
cerevisiae Rad50*Mre11 complex.. J Biol Chem.

[17] Hopfner KP, Karcher A, Shin DS, Craig L, Arthur LM, Carney JP, Tainer JA (2000). Structural biology of Rad50 ATPase: ATP-driven conformational
control in DNA double-strand break repair and the ABC-ATPase
superfamily.. Cell.

[18] de Jager M, van Noort J, van Gent DC, Dekker C, Kanaar R, Wyman C (2001). Human Rad50/Mre11 is a flexible complex that can tether DNA
ends.. Mol Cell.

[19] Hopfner KP, Karcher A, Craig L, Woo TT, Carney JP, Tainer JA (2001). Structural biochemistry and interaction architecture of the DNA
double-strand break repair Mre11 nuclease and Rad50-ATPase.. Cell.

[20] Hopfner K-P, Craig L, Moncalian G, Zinkel RA, Usui T, Owen BAL, Karcher A, Henderson B, Bodmer J-L, McMurray CT, Carney JP, Petrini JHJ, Tainer JA (2002). The Rad50 zinc-hook is a structure joining Mre11 complexes in DNA
recombination and repair.. Nature.

[21] Moncalian G, Lengsfeld B, Bhaskara V, Hopfner K-P, Karcher A, Alden E, Tainer JA, Paull TT (2004). The Rad50 signature motif: essential to ATP binding and
biological function.. J Mol Biol.

[22] Moreno-Herrero F, de Jager M, Dekker NH, Kanaar R, Wyman C, Dekker C (2005). Mesoscale conformational changes in the DNA-repair complex
Rad50/Mre11/Nbs1 upon binding DNA.. Nature.

[23] Lammens K, Bemeleit DJ, Möckel C, Clausing E, Schele A, Hartung S, Schiller CB, Lucas M, Angermüller C, Söding J, Strässer K, Hopfner KP (2011). The Mre11:Rad50 structure shows an ATP-dependent molecular clamp
in DNA double-strand break repair.. Cell.

[24] Lim HS, Kim JS, Park YB, Gwon GH, Cho Y (2011). Crystal structure of the Mre11-Rad50-ATPgS complex: understanding
the interplay between Mre11 and Rad50.. Genes Dev.

[25] Williams GJ, Williams RS, Williams JS, Moncalian G, Arvai AS, Limbo O, Guenther G, SilDas S, Hammel M, Russell P, Tainer JA (2011). ABC ATPase signature helices in Rad50 link nucleotide state to
Mre11 interface for DNA repair.. Nat Struct Mol Biol.

[26] Möckel C, Lammens K, Schele A, Hopfner KP (2012). ATP driven structural changes of the bacterial Mre11:Rad50
catalytic head complex.. Nucleic Acids Res.

[27] Bressan DA, Baxter BK, Petrini JH (1999). The Mre11-Rad50-Xrs2 protein complex facilitates homologous
recombination-based double-strand break repair in Saccharomyces
cerevisiae.. Mol Cell Biol.

[28] Chen L, Trujillo K, Ramos W, Sung P, Tomkinson AE (2001). Promotion of Dnl4-catalyzed DNA end-joining by the
Rad50/Mre11/Xrs2 and Hdf1/Hdf2 complexes.. Mol Cell.

[29] Lobachev K, Vitriol E, Stemple J, Resnick MA, Bloom K (2004). Chromosome fragmentation after induction of a double-strand break
is an active process prevented by the RMX repair complex.. Curr Biol.

[30] Wiltzius JJW, Hohl M, Fleming JC, Petrini JHJ (2005). The Rad50 hook domain is a critical determinant of Mre11 complex
functions.. Nat Struct Mol Biol.

[31] Hohl M, Kwon Y, Galván SM, Xue X, Tous C, Aguilera A, Sung P, Petrini JHJ (2011). The Rad50 coiled-coil domain is indispensable for Mre11 complex
functions.. Nat Struct Mol Biol.

[32] Seifert FU, Lammens K, Stoehr G, Kessler B, Hopfner KP (2016). Structural mechanism of ATP-dependent DNA binding and DNA end
bridging by eukaryotic Rad50.. EMBO J.

[33] Tsukamoto Y, Mitsuoka C, Terasawa M, Ogawa H, Ogawa T (2005). Xrs2p regulates Mre11p translocation to the nucleus and plays a
role in telomere elongation and meiotic recombination.. Mol Biol Cell.

[34] Lloyd J, Chapman JR, Clapperton JA, Haire LF, Hartsuiker E, Li J, Carr AM, Jackson SP, Smerdon SJ (2009). A supramodular FHA/BRCT-repeat architecture mediates Nbs1 adaptor
function in response to DNA damage.. Cell.

[35] Nakada D, Matsumoto K, Sugimoto K (2003). ATM-related Tel1 associates with double-strand breaks through an
Xrs2-dependent mechanism.. Genes Dev.

[36] Liu Y, Sung S, Kim Y, Li F, Gwon G, Jo A, Kim AK, Kim T, Song OK, Lee SE, Cho Y (2016). ATP-dependent DNA binding, unwinding, and resection by the
Mre11/Rad50 complex.. EMBO J.

[37] Deshpande RA, Williams GJ, Limbo O, Williams RS, Kuhnlein J, Lee J-H, Classen S, Guenther G, Russell P, Tainer JA, Paull TT (2014). ATP-driven Rad50 conformations regulate DNA tethering, end
resection, and ATM checkpoint signaling.. EMBO J.

[38] Majka J, Alford B, Ausio J, Finn RM, McMurray CT (2012). ATP hydrolysis by RAD50 protein switches MRE11 enzyme from
endonuclease to exonuclease.. J Biol Chem.

[39] Wotton D, Shore D (1997). A novel Rap1p-interacting factor, Rif2p, cooperates with Rif1p to
regulate telomere length in Saccharomyces cerevisiae.. Genes Dev.

[40] Levy DL, Blackburn EH (2004). Counting of Rif1p and Rif2p on Saccharomyces cerevisiae telomeres
regulates telomere length.. Mol Cell Biol.

[41] Hirano Y, Fukunaga K, Sugimoto K (2009). Rif1 and Rif2 inhibit localization of Tel1 to DNA
ends.. Mol Cell.

[42] Martina M, Clerici M, Baldo V, Bonetti D, Lucchini G, Longhese MP (2012). A balance between Tel1 and Rif2 activities regulates nucleolytic
processing and elongation at telomeres.. Mol Cell Biol.

[43] Cassani C, Gobbini E, Wang W, Niu H, Clerici M, Sung P, Longhese MP (2016). Tel1 and Rif2 regulate MRX functions in end-tethering and repair
of DNA double-strand breaks.. PLoS Biol.

[44] Marcand S, Pardo B, Gratias A, Cahun S, Callebaut I (2008). Multiple pathways inhibit NHEJ at telomeres.. Genes Dev.

[45] Ivanov EL, Sugawara N, White CI, Fabre F, Haber JE (1994). Mutations in XRS2 and RAD50 delay but do not prevent mating-type
switching in Saccharomyces cerevisiae.. Mol Cell Biol.

[46] Clerici M, Mantiero D, Lucchini G, Longhese MP (2005). The Saccharomyces cerevisiae Sae2 protein promotes resection and
bridging of double strand break ends.. J Biol Chem.

[47] Clerici M, Mantiero D, Lucchini G, Longhese MP (2006). The Saccharomyces cerevisiae Sae2 protein negatively regulates
DNA damage checkpoint signalling.. EMBO Rep.

[48] Moreau S, Morgan EA, Symington LS (2001). Overlapping functions of the Saccharomyces cerevisiae Mre11, Exo1
and Rad27 nucleases in DNA metabolism.. Genetics.

[49] Lobachev KS, Gordenin DA, Resnick MA (2002). The Mre11 complex is required for repair of hairpin-capped
double-strand breaks and prevention of chromosome
rearrangements.. Cell.

[50] Yu J, Marshall K, Yamaguchi M, Haber JE, Weil CF (2004). Microhomology-dependent end joining and repair of
transposon-induced DNA hairpins by host factors in Saccharomyces
cerevisiae.. Mol Cell Biol.

[51] Rattray AJ, Shafer BK, Neelam B, Strathern JN (2005). A mechanism of palindromic gene amplification in Saccharomyces
cerevisiae.. Genes Dev.

[52] Cao L, Alani E, Kleckner N (1990). A pathway for generation and processing of double-strand breaks
during meiotic recombination in S. cerevisiae.. Cell.

[53] Alani E, Padmore R, Kleckner N (1990). Analysis of wild-type and rad50 mutants of yeast suggests an
intimate relationship between meiotic chromosome synapsis and
recombination.. Cell.

[54] Nairz K, Klein F (1997). mre11S, a yeast mutation that blocks double-strand-break
processing and permits nonhomologous synapsis in meiosis.. Genes Dev.

[55] Prinz S, Amon A, Klein F (1997). Isolation of COM1, a new gene required to complete meiotic
double-strand break-induced recombination in Saccharomyces
cerevisiae.. Genetics.

[56] Tsubouchi H, Ogawa H (1998). A novel mre11 mutation impairs processing of double-strand breaks
of DNA during both mitosis and meiosis.. Mol Cell Biol.

[57] Hopkins BB, Paull TT (2008). The P. furiosus Mre11/Rad50 complex promotes 5’ strand resection at a DNA
double-strand break.. Cell.

[58] Williams RS, Moncalian G, Williams JS, Yamada Y, Limbo O, Shin DS, Groocock LM, Cahill D, Hitomi C, Guenther G, Moiani D, Carney JP, Russell P, Tainer JA (2008). Mre11 dimers coordinate DNA end bridging and nuclease processing
in double-strand-break repair.. Cell.

[59] Cannavo E, Cejka P (2014). Sae2 promotes dsDNA endonuclease activity within Mre11-Rad50-Xrs2
to resect DNA breaks.. Nature.

[60] Keeney S, Kleckner N (1995). Covalent protein-DNA complexes at the 5’ strand termini of
meiosis-specific double-strand breaks in yeast.. Proc Natl Acad Sci USA.

[61] Neale MJ, Pan J, Keeney S (2005). Endonucleolytic processing of covalent protein-linked DNA
double-strand breaks.. Nature.

[62] Hartsuiker E, Mizuno K, Molnar M, Kohli J, Ohta K, Carr AM (2009). Ctp1/CtIP and Rad32/Mre11 nuclease activity are required for
Rec12/Spo11 removal, but Rec12/Spo11 removal is dispensable for other
MRN-dependent meiotic functions.. Mol Cell Biol.

[63] Milman N, Higuchi E, Smith GR (2009). Meiotic DNA double-strand break repair requires two nucleases,
MRN and Ctp1, to produce a single size class of Rec12
(Spo11)-oligonucleotide complexes.. Mol Cell Biol.

[64] Hartsuiker E, Neale MJ, Carr AM (2009). Distinct requirements for the Rad32(Mre11) nuclease and
Ctp1(CtIP) in the removal of covalently bound topoisomerase I and II from
DNA.. Mol Cell.

[65] Henner WD, Grunberg SM, Haseltine WA (1983). Enzyme action at 3’ termini of ionizing radiation-induced DNA
strand breaks.. J Biol Chem.

[66] Barker S, Weinfeld M, Zheng J, Li L, Murray D (2005). Identification of mammalian proteins cross-linked to DNA by
ionizing radiation.. J Biol Chem.

[67] Mimitou EP, Symington LS (2008). Sae2, Exo1 and Sgs1 collaborate in DNA double-strand break
processing.. Nature.

[68] Zhu Z, Chung W-H, Shim EY, Lee SE, Ira G (2008). Sgs1 helicase and two nucleases Dna2 and Exo1 resect DNA
double-strand break ends.. Cell.

[69] Lee BI, Wilson DM (1999). The RAD2 domain of human exonuclease 1 exhibits 5’ to 3’
exonuclease and flap structure-specific endonuclease
activities.. J Biol Chem.

[70] Tran PT, Erdeniz N, Dudley S, Liskay RM (2002). Characterization of nuclease-dependent functions of Exo1p in
Saccharomyces cerevisiae.. DNA Repair.

[71] Cannavo E, Cejka P, Kowalczykowski SC (2013). Relationship of DNA degradation by Saccharomyces cerevisiae
exonuclease 1 and its stimulation by RPA and Mre11-Rad50-Xrs2 to DNA end
resection.. Proc Natl Acad Sci USA.

[72] Cejka P, Cannavo E, Polaczek P, Masuda-Sasa T, Pokharel S, Campbell JL, Kowalczykowski SC (2010). DNA end resection by Dna2-Sgs1-RPA and its stimulation by
Top3-Rmi1 and Mre11-Rad50-Xrs2.. Nature.

[73] Niu H, Chung W-H, Zhu Z, Kwon Y, Zhao W, Chi P, Prakash R, Seong C, Liu D, Lu L, Ira G, Sung P (2010). Mechanism of the ATP-dependent DNA end-resection machinery from
Saccharomyces cerevisiae.. Nature.

[74] Nimonkar AV, Genschel J, Kinoshita E, Polaczek P, Campbell JL, Wyman C, Modrich P, Kowalczykowski SC (2011). BLM-DNA2-RPA-MRN and EXO1-BLM-RPA-MRN constitute two DNA end
resection machineries for human DNA break repair.. Genes Dev.

[75] Shim EY, Chung W-H, Nicolette ML, Zhang Y, Davis M, Zhu Z, Paull TT, Ira G, Lee SE (2010). Saccharomyces cerevisiae Mre11/Rad50/Xrs2 and Ku proteins
regulate association of Exo1 and Dna2 with DNA breaks.. EMBO J.

[76] Zierhut C, Diffley JF (2008). Break dosage, cell cycle stage and DNA replication influence DNA
double strand break response.. EMBO J.

[77] Huertas P, Cortés-Ledesma F, Sartori AA, Aguilera A, Jackson SP (2008). CDK targets Sae2 to control DNA-end resection and homologous
recombination.. Nature.

[78] Chen X, Niu H, Chung WH, Zhu Z, Papusha A, Shim EY, Lee SE, Sung P, Ira G (2011). Cell cycle regulation of DNA double-strand break end resection by
Cdk1-dependent Dna2 phosphorylation.. Nat Struct Mol Biol.

[79] Aylon Y, Liefshitz B, Kupiec M (2004). The CDK regulates repair of double-strand breaks by homologous
recombination during the cell cycle.. EMBO J.

[80] Ira G, Pellicioli A, Balijja A, Wang X, Fiorani S, Carotenuto W, Liberi G, Bressan D, Wan L, Hollingsworth NM, Haber JE, Foiani M (2004). DNA end resection, homologous recombination and DNA damage
checkpoint activation require CDK1.. Nature.

[81] Clerici M, Mantiero D, Guerini I, Lucchini G, Longhese MP (2008). The Yku70-Yku80 complex contributes to regulate double-strand
break processing and checkpoint activation during the cell
cycle.. EMBO Rep.

[82] Garcia V, Phelps SE, Gray S, Neale MJ (2011). Bidirectional resection of DNA double-strand breaks by Mre11 and
Exo1.. Nature.

[83] Lempiäinen H, Halazonetis TD (2009). Emerging common themes in regulation of PIKKs and
PI3Ks.. EMBO J.

[84] Bosotti R, Isacchi A, Sonnhammer EL (2000). FAT: a novel domain in PIK-related kinases.. Trends Biochem Sci.

[85] Shiloh Y, Ziv Y (2013). The ATM protein kinase: regulating the cellular response to
genotoxic stress, and more.. Nat Rev Mol Cell Biol.

[86] Paull TT (2015). Mechanisms of ATM Activation.. Annu Rev Biochem.

[87] Meyn MS (1995). Ataxia-telangiectasia and cellular responses to DNA
damage.. Cancer Res.

[88] Lavin MF, Shiloh Y (1997). The genetic defect in ataxia-telangiectasia.. Annu Rev Immunol.

[89] McKinnon PJ (2004). ATM and ataxia telangiectasia.. EMBO Rep.

[90] Stewart GS, Maser RS, Stankovic T, Bressan DA, Kaplan MI, Jaspers NG, Raams A, Byrd PJ, Petrini JH, Taylor AM (1999). The DNA double-strand break repair gene hMRE11 is mutated in
individuals with an ataxia-telangiectasia-like disorder.. Cell.

[91] Taylor AMR, Groom A, Byrd PJ (2004). Ataxia-telangiectasia-like disorder (ATLD)-its clinical
presentation and molecular basis.. DNA Repair.

[92] Uziel T, Lerenthal Y, Moyal L, Andegeko Y, Mittelman L, Shiloh Y (2003). Requirement of the MRN complex for ATM activation by DNA
damage.. EMBO J.

[93] Falck J, Coates J, Jackson SP (2005). Conserved modes of recruitment of ATM, ATR and DNA-PKcs to sites
of DNA damage.. Nature.

[94] You Z, Chahwan C, Bailis J, Hunter T, Russell P (2005). ATM activation and its recruitment to damaged DNA require binding
to the C terminus of Nbs1.. Mol Cell Biol.

[95] Fukunaga K, Kwon Y, Sung P, Sugimoto K (2011). Activation of protein kinase Tel1 through recognition of
protein-bound DNA ends.. Mol Cell Biol.

[96] Lee JH, Paull TT (2004). Direct activation of the ATM protein kinase by the
Mre11/Rad50/Nbs1 complex.. Science.

[97] Lee JH, Paull TT (2005). ATM activation by DNA double-strand breaks through the
Mre11-Rad50-Nbs1 complex.. Science.

[98] Dupré A, Boyer-Chatenet L, Gautier J (2006). Two-step activation of ATM by DNA and the Mre11-Rad50-Nbs1
complex.. Nat Struct Mol Biol.

[99] Lustig AJ, Petes TD (1986). Identification of yeast mutants with altered telomere
structure.. Proc Natl Acad Sci USA.

[100] Greenwell PW, Kronmal SL, Porter SE, Gassenhuber J, Obermaier B, Petes TD (1995). TEL1, a gene involved in controlling telomere length in
S. cerevisiae, is homologous to the human ataxia telangiectasia gene.. Cell.

[101] Morrow DM, Tagle DA, Shiloh Y, Collins FS, Hieter P (1995). TEL1, an S. cerevisiae homolog of the human gene mutated in
ataxia telangiectasia, is functionally related to the yeast checkpoint gene
MEC1.. Cell.

[102] Wellinger RJ, Zakian VA (2012). Everything you ever wanted to know about Saccharomyces cerevisiae
telomeres: beginning to end.. Genetics.

[103] Ritchie KB, Petes TD (2000). The Mre11p/Rad50p/Xrs2p complex and the Tel1p function in a
single pathway for telomere maintenance in yeast.. Genetics.

[104] Hector RE, Shtofman RL, Ray A, Chen B-R, Nyun T, Berkner KL, Runge KW (2007). Tel1p preferentially associates with short telomeres to stimulate
their elongation.. Mol Cell.

[105] Sabourin M, Tuzon CT, Zakian VA (2007). Telomerase and Tel1p preferentially associate with short
telomeres in S. cerevisiae.. Mol Cell.

[106] Mantiero D, Clerici M, Lucchini G, Longhese MP (2007). Dual role for Saccharomyces cerevisiae Tel1 in the checkpoint
response to double-strand breaks.. EMBO Rep.

[107] Shiotani B, Zou L (2009). Single-stranded DNA orchestrates an ATM-to-ATR switch at DNA
breaks.. Mol Cell.

[108] Usui T, Ogawa H, Petrini JH (2001). A DNA damage response pathway controlled by Tel1 and the Mre11
complex.. Mol Cell.

[109] Duursma AM, Driscoll R, Elias JE, Cimprich KA (2013). A role for the MRN complex in ATR activation via TOPBP1
recruitment.. Mol Cell.

[110] Beyer T, Weinert T (2014). Mec1 and Tel1: an arresting dance of resection.. EMBO J.

[111] Paciotti V, Clerici M, Lucchini G, Longhese MP (2000). The checkpoint protein Ddc2, functionally related to S. pombe
Rad26, interacts with Mec1 and is regulated by Mec1-dependent
phosphorylation in budding yeast.. Genes Dev.

[112] Rouse J, Jackson SP (2002). Lcd1p recruits Mec1p to DNA lesions in vitro and in
vivo.. Mol Cell.

[113] Zou L, Elledge SJ (2003). Sensing DNA damage through ATRIP recognition of RPA-ssDNA
complexes.. Science.

[114] Nakada D, Hirano Y, Tanaka Y, Sugimoto K (2005). Role of the C terminus of Mec1 checkpoint kinase in its
localization to sites of DNA damage.. Mol Biol Cell.

[115] Hohl M, Kochańczyk T, Tous C, Aguilera A, Krężel A, Petrini JH (2015). Interdependence of the rad50 hook and globular domain
functions.. Mol Cell.

[116] Nassif N, Penney J, Pal S, Engels WR, Gloor GB (1994). Efficient copying of nonhomologous sequences from ectopic sites
via P-element-induced gap repair.. Mol Cell Biol.

[117] Ferguson DO, Holloman WK (1996). Recombinational repair of gaps in DNA is asymmetric in Ustilago
maydis and can be explained by a migrating D-loop model.. Proc Natl Acad Sci USA.

[118] Pâques F, Leung WY, Haber JE (1998). Expansions and contractions in a tandem repeat induced by
double-strand break repair.. Mol Cell Biol.

